# Electrospun Polyphosphate Coacervate Glass Fibers in the System P_2_O_5_–CaO–MgO–Na_2_O–Fe_2_O_3_ for Wound Healing

**DOI:** 10.1021/acsomega.4c09366

**Published:** 2025-03-17

**Authors:** Jack Humphray, Agron Hoxha, Eveliny Tomás Nery, Charlotte Berry, Mónica Felipe-Sotelo, Holly Wilkinson, Matthew Hardman, Jorge Gutiérrez-Merino, Daniela Carta

**Affiliations:** † School of Chemistry and Chemical Engineering, University of Surrey, Guildford GU2 7XH, U.K.; ‡ School of Biosciences and Medicine, University of Surrey, Guildford GU2 7XH, U.K.; § Centre for Biomedicine, Hull York Medical School, University of Hull, Hull HU6 7RX, U.K.; ∥ Skin Research Centre, Hull York Medical School, University of York, York YO10 5DD, U.K.

## Abstract

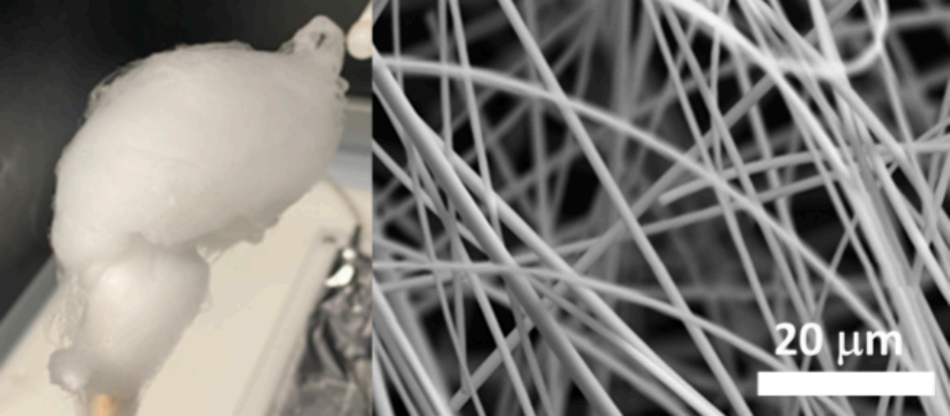

This study investigates a series of phosphate-glass fibers (PGFs) in the system P_2_O_5_–CaO–MgO–Na_2_O–Fe_2_O_3_ with various Fe contents (0, 0.1, 0.5, 1, and 2 wt %) prepared via electrospinning of polyphosphate coacervate gels. This method is preferable over the traditional high-temperature melt-spinning technique used for PGF production as it represents a more cost-effective and sustainable route. Structural analysis performed via Fourier transform Infrared spectroscopy shows that PGFs are mainly formed by polyphosphate chains containing Q^1^ and Q^2^ units. Thermal analysis demonstrates that the amorphous nature of the PGFs can be preserved up to calcination temperatures in the range 450–520 °C, with crystallization temperatures increasing with the iron content. Dissolution studies were performed by immersing the PGFs in deionized water and analyzing the species released (P, Ca, Mg, Fe, and Na) via microwave plasma atomic emission spectroscopy at regular intervals up to 72 hours (h). Results show that both iron and phosphate anion release increases with iron loading, suggesting that the phosphate network is weakened by an increasing amount of iron. Given that PGFs are particularly advantageous in wound healing due to their fibrous morphology, their cytocompatibility was assessed by seeding human keratinocytes (HaCaTs) in contact with the dissolution products of PGFs after 24 h of immersion at three different ratios of dissolution products to cell medium (1:100, 3:100, and 5:100). No cytotoxicity was observed for any of the ratios studied. Moreover, the dissolution products of some PGFs resulted in an enhanced growth of HaCaTs, with the best result being observed when using dissolution products from PGFs containing 0.1 wt % of Fe and a dissolution product-cell medium ratio of 5:100. Dissolution products from PGFs with an Fe content up to 0.5 wt % have also demonstrated antibacterial activity against the bacterium *Escherichia coli* (*E. coli*). A preliminary test on the efficacy of PGFs in wound healing via *ex vivo* studies on human skin has demonstrated that the PGFs in direct contact with the wound promote 84% wound closure.

## 1 Introduction

Phosphate-based glasses (PGs) have great potential as biomaterials for controlled, sustainable delivery of therapeutic species.^1,2^ Being bioresorbable, they dissolve in body fluids over time, releasing species capable of inducing tissue regeneration and/or antibacterial activity.^3^ The most common PG system investigated as a bioresorbable biomaterial is based on the ternary system P_2_O_5_–CaO–Na_2_O with the addition of various therapeutic metallic ions (TMIs). The release upon dissolution of specific TMIs has been shown to bestow the glass with osteogenic (Ca^2+^), hemostatic (e.g., Ca^2+^, Mg^2+^, Fe^2+/3+^), and antibacterial (e.g., Ag^+^, Cu^2+^, Ga^3+^, Zn^2+^) properties.^4^ The release properties of PGs can be controlled and tailored to the specific application by changing the CaO/Na_2_O ratio or by adding additional oxides.^5^ The use of inorganic-oxide glasses for soft tissue regeneration is a very timely topic.^6^ Despite having been mainly studied for hard tissue regeneration,^3,4,7^ PGs have also been recently proposed for soft tissue generation.^8−10^

Inorganic-oxide glass fibers have been found to have many advantages over conventional wound matrices. Their high surface area-to-volume ratio and open porosity promote gaseous exchange, removal of exudate, and cell migration, while surface hydroxyl groups permit functionalization.^11^ Moreover, the nanoscale architecture of fibers, similar to the extracellular matrix that is composed of collagen fibers, is well suited to wound healing. Fibers can be easily packed into tight and complex defects and manufactured into mats and yarns for wound dressings.^12^

Silicate-based glass electrospun fibers have been recently investigated for their potential applications in wound healing.^13^ Commonly, silicate-glasses are combined with a polymeric binder to obtain the desired viscosity range that allows electrospinning (ES). Poly(D, L-lactic acid) has been used in addition to Bioglass to be used as a composite coating on surgical sutures,^14^ and poly(ε-caprolactone) has been used as a polymeric agent for ES of melt-derived, silicate-glasses containing Co, Mg, Sr, and Zn for wound-healing.^15,16^

Norris *et al*.^13^ investigated glass fibrous scaffolds in the SiO_2_–CaO system prepared via sol–gel for wound-healing applications. The polymer binding agent (polyvinylbutyral, Butvar-98) was added to the inorganic sol–gel solutions, enabling ES prior to bioactive glass network formation. The polymer was then removed by calcination at 600 °C. However, this caused crystallization issues of some of the systems prepared. The same binding agent Butvar-98 was used to synthesize a SiO_2_–CaO system with added 2 mol % of silver for tackling the bacterium **Pseudomonas aeruginosa*.* Addition of a polymer to the silicate glass powders prior to ES often causes issues as the glass powders might not be homogeneously distributed in the polymer, inducing stress or formation of aggregates. Phosphate-glass fibers (PGFs) prepared in this work overcome this issue as the fully injectable gels contain the bioactive components (P, Ca, and Na) well dispersed in a colloidal system. Additional active species, such as TMIs, can be added directly into the gel, achieving uniform distribution.

While some work has been done on the application of silicate-based glass fibers to soft tissue engineering, very little work has been done on the use of PGFs for biomedical applications, in particular for wound healing.

Most PGFs presented in the literature are prepared via the traditional melt-spinning (MS) technique where oxide powders are melted at temperatures >1100 °C followed by drawing fibers at very fast cooling rates.^8−10^ However, MS is incompatible with thermal-sensitive molecules, fails to deliver high surface areas and porosity, and may cause reduction of ions in the melt and loss of volatile phosphorus. Despite these limitations, PGFs prepared via MS have been investigated for the engineering of fibrous soft tissues (muscles, ligaments, and nerve conduits) as a guide for cell growth and migration, important steps in wound healing.^9,10,17^ Literature on the use of PGFs for wound treatment is limited to a few studies on melt-derived fibers doped with Ga^3+^ and Ce^4+^.^18^ In this work, an alternative, room-temperature, water-based manufacturing process called coacervation was used to prepare polyphosphate-based gels which were subsequently drawn into PGFs via ES.^19^ Coacervate gels are formed by the addition of 2+ cations (e.g., Ca^2+^ and Mg^2+^) to a sodium polyphosphate aqueous solution.^20^ The electrostatic interactions between the long polyphosphate chains and bivalent cations lead to the formation of the coacervate gels. The gels are then loaded into a syringe, injected into a nozzle, and upon application of a high voltage (10–20 kV), electrospun, forming PGFs that are then deposited on a screen collector or on a rotating drum. ES produces cotton-like fibers of improved conformability to complex wound topographies. Unlike ES of most polymers used in wound dressings (*e.g.*, chitosan, cellulose, or gelatin), ES of phosphate-based coacervate gels does not require toxic or acidic solvents. This method has many advantages over MS as both preparation of the precursors and ES are performed at room temperature in mild conditions, allowing temperature-sensitive molecules to be incorporated into the gels. The first example of PGFs produced via ES of coacervate gels in the system P_2_O_5_–CaO–Na_2_O–(CuO)_*x*_ (*x* = 0, 1, 3, and 5 mol %) was recently presented by Foroutan *et al.*.^20^ More recently, Hoxha *et al.* have presented Ag, Zn, and Fe loaded PGFs for wound healing promotion.^21^ Various ions have been found to be effective in promoting specific stages of wound healing (hemostasis, inflammation, proliferation, and remodelling). In particular, a crucial role has been reported to be played by Ca^2+^ and Mg^2+^ in the coagulation cascade (hemostasis).^22,23^

It has been recently shown that diabetic murine wounds display significantly reduced iron levels; it has been therefore suggested that iron ions might have a role in facilitating the remodelling stage of wound healing.^24,25^ In addition, iron ions have been reported to cause bacterial death through oxidative damage.^26^

In this work, we have investigated PGFs in the system P_2_O_5_–CaO–MgO–Na_2_O–Fe_2_O_3_ (0, 0.1, 0.5, 1, and 2 wt % of Fe) manufactured via ES of coacervate gels for wound-healing applications. Structural and morphological investigation of the PGFs along with dissolution studies, cytocompatibility of human keratinocytes (HaCaTs), and antibacterial activity against *E. coli* have been discussed. Preliminary data on *ex vivo* wound models on the P_2_O_5_–CaO–MgO–Na_2_O system are also presented.

## 2 Experimental Section

### 2.1 Synthesis of Coacervate Gels

**Figure 1:**
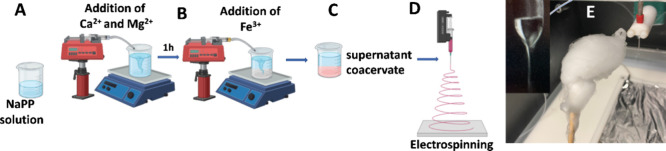
Schematic of ES of coacervate gels for PGF production: (A) Addition of Ca^2+^ and Mg^2+^ to the NaPP solution. (B) Addition of Fe^3+^ to the solution prepared in (A). (C) Formation of the coacervate gel. (D) Electrospinning of the coacervate gel. (E) Image of the electrospun fibers (inset: image of the Taylor cone).

The process used for the synthesis of PGFs is illustrated in Figure 1.

For the synthesis of P_2_O_5_–CaO–MgO–Na_2_O PGFs, 10 mL of a 1 M aqueous solution of magnesium nitrate hexahydrate [Mg (NO_3_)_2_·6H_2_O; Sigma-Aldrich 99%] and 10 mL of a 1 M aqueous solution of calcium nitrate tetrahydrate [Ca(NO_3_)_2_·4H_2_O; Sigma-Aldrich 99%] were added dropwise via a syringe pump (20 mL h ^–1^) to 20 mL of a 0.016 M aqueous solution of sodium polyphosphate [Na(PO_3_)_*n*_, *n* ∼ 25, Merck 99.0%] (Figure 1A) under vigorous stirring for 1 h.

For the synthesis of P_2_O_5_–CaO–MgO–Na_2_O–Fe_2_O_3_ PGFs containing 0.1, 0.5, 1, and 2 wt % of Fe, 0.12, 0.6, 1.2, and 3.6 mL of a 2 M aqueous solution of iron nitrate nonahydrate [Fe(NO_3_)_3_·9H_2_O; Sigma-Aldrich 98%] were added to the solution prepared as above, and the solution was stirred for 1 h (Figure 1B). This resulted in the formation of two layers: a clear, aqueous layer (top) and a more viscous “gel-like” opaque bottom layer (coacervate) containing the polyphosphate chains (Figure 1C). Images of selected coacervate gels are reported in Figure SI-1. After leaving the mixture to settle for 1 h, the top layer was discarded, and the bottom coacervate layer was left to settle for 24 h at room temperature. If additional supernatant had separated during this time, it was also discarded. PGFs were then produced via ES of the polyphosphate coacervate (Figure 1D) using a Spraybase electrospinner (Kildare, Ireland). The gels were inserted in a syringe and injected using a syringe pump into an 18-gauge nozzle using a flow rate in the range 0.75–2 mL h ^–1^ forming a drop at its tip. By application of a 15–20 kV potential difference between the nozzle and the metallic collector, the electrical field distorts the drop into a conical shape, known as a Taylor cone (inset of Figure 1E). The precursors were then ejected from the nozzle, and the cotton-like PGFs were then collected from the metal collector as a bundle (Figure 1E). Flow rate and voltage were adjusted depending on the composition. PGF compositions were chosen based on previous works on bulk PGs prepared via MQ and SG; PGs with similar P and Ca contents were shown to have good biocompatibility and bioactivity.^26^ Samples will be hereafter named PGF-und (fibers without Fe_2_O_3_) and PGF-X, where X is the weight (wt) % of Fe.

### 2.2 Characterization

Powder X-ray Diffraction (XRD) was carried out with a PANalytical X’pert powder X-ray diffractometer using Cu K_α_ radiation (λ = 1.54 Å) in transmission mode. The samples were analyzed over a 2ϑ angle in the range 15°–85° with a step size of 0.0525° and a collection time of 1.8 s per step.

Fourier Transform-InfraRed (FT-IR) spectroscopy was performed using a Perkin Elmer Spectrometer BXII equipped with a Specac Quest single reflective attenuated total reflectance accessory. 64 scans per sample were collected in the range 800–4000 cm^–1^ at a resolution of 2 cm^–1^.

Raman spectra were collected using a ThermoFisher Scientific DXR Raman spectrometer with a 532 nm laser at 10 mW in continuous mode. Each spectrum was scanned in the range 620–1400 cm^–1^ with an integration time of 0.5 s and 10 accumulations.

Thermogravimetric analysis (TGA) and differential scanning calorimetry (DSC) were both performed with a SDT650 instrument (TA Instruments) from 25 to 800 °C with a heating rate of 10 °C per minute in air.

Scanning electron microscopy (SEM) images and energy-dispersive X-ray (EDX) spectra were collected using a Thermo Fisher Apreo S instrument with an accelerating voltage of 15 kV at a variable probe current. Samples were mounted with conductive carbon tape onto an aluminum stub and carbon coated before observation to avoid charging. A Thermo Fisher Quasor II detector was used for the EDX spectra collection. MIPAR image analysis software (version 4.0.1) was utilized to measure the average thickness of PGFs.^27^

### 2.3 Release Studies

5 mg of each PGF were immersed into 5 mL of deionized (DI) water (Veolia Water, Elga Centra, resistivity 18.2MΩ·cm) in separate vials and left in an incubator shaker at 37 °C for 3, 6, 24, 48, and 72 h under stirring at 100 rpm. Release tests in DI water are commonly performed on the PGs used for tissue repair,^28^ and they are also useful in assessing the applicability of PGs in different fields (e.g., optical).^29^ Each dissolution test was performed in triplicate. Following centrifugation at 4800 rpm for 5 min, the supernatant was filtered through a 0.45 μm unit (Millipore filter unit, Millex GP) and stored at 4 °C before analysis. Quantitative analysis of degradation products was performed via microwave plasma atomic emission spectroscopy (MP-AES) using an Agilent MP-AES 4210 instrument. Concentrations of phosphorus, sodium, calcium, magnesium and iron were measured using standards of 0.5, 1, 2, 5, 10, 25, 50, 75, and 100 μg mL^–1^ prepared from commercial 1000 μg mL^–1^ stock solutions (PlasmaCAL). All samples and standards were diluted with 2% HNO_3_ (Trace Metal Analysis grade, Fisher Scientific). The signals were blank-corrected using 2% HNO_3_, and the analyte emission signals for each element (Na 588.95 nm, Ca 422.67 nm, P 213.618 nm, Mg 285.213 nm and Fe 371.993 nm) were normalized by ratioing the beryllium internal standard intensity (5 μg mL^–1^, 234.86 and 313.04 nm) for correction of sensitivity drift during analysis. The instrument was calibrated daily with freshly prepared standards.

### 2.4 Antibacterial Activity

Antibacterial activity of the PGFs was tested by incubating *E. coli* K12 strains with the dissolution products of PGFs after 24 h of immersion in DI water. Prior to incubation with PGF’s dissolution products, bacterial strains were grown in Tryptic soy broth (TSB) in an incubator shaker (37 °C, 250 rpm) until the cultures reached the mid-exponential phase of growth. Bacteria were then diluted to an optical density of 0.05 at 600 nm (OD600) in 96-well plates with TSB containing the 24 h dissolution products of PGF (5:100 ratio) and incubated at 37 °C without shaking. After 24 h, the OD600 of each well was measured using a CLARIOstar plate reader (BMG LABTECH). The values obtained were corrected with a blank containing only TSB, and an ordinary one-way ANOVA with Dunnet’s multiple comparison tests was performed for all samples to determine statistically significant differences between samples (*p ≤ 0.05; **p ≤ 0.01; ****p ≤ 0.0001).

### 2.5 Cytotoxicity Testing

3-(4, 5-Dimethylthiazolyl-2)-2,5-diphenyltetrazolium bromide (MTT) tests were performed for investigate the cytocompatibility of PGF dissolution products. An immortalized cell line of human keratinocytes (HaCaTs in vitro spontaneously transformed keratinocytes from histologically normal skin, AddexBio, Catalog Number T0020001, San Diego, US) were diluted to a concentration of approximately 10^5^ cells mL^–1^ in Dulbecco’s modified Eagle medium (DMEM, Gibco) prepared with l-Glutamine, streptomycin/penicillin, 2% FBS, and 0.4 mM CaCl_2_ and seeded in 96-well tissue culture treated plates. HaCaTs are frequently used in skin research as a reliable *in vitro* model of immunological and inflammatory response.^30^

The cells were then incubated at 37 °C in an atmosphere containing 5% CO_2_ and allowed to grow for 24 h. A medium containing the 24 h dissolution products of PGF-und, PGF-Fe0.1, PGF-Fe0.5, PGF-Fe1, and PGF-Fe2 was then added to each well (1, 3, and 5:100 ratios) in triplicate, and HaCaTs were incubated for 24 h. Positive controls consisted of HaCaTs incubated with medium only (no dissolution products).

Then, 12 μL of MTT reagent at 1 mg mL^–1^ (rhiazolyl blue tetrazolium bromide, Sigma-Aldrich) were added to each well, and the plate was incubated for 3 h. The medium was aspirated, and 100 μL of dimethylsulfoxide (DMSO 99,9%, Sigma-Aldrich) was added to each well. The absorbance at 570 nm of the cultures was measured using a microplate reader (Fluostar-Omega, BMG LabTech). The percentage of metabolically active HaCaTs was calculated by comparing the average absorbance at 570 nm of the positive control to the absorbance of the respective treatments. An ordinary one-way ANOVA with Dunnett’s multiple comparison tests was applied as per the antibacterial activity (*p ≤ 0.05; **p ≤ 0.01; ****p ≤ 0.0001).

### 2.6 *Ex Vivo* Wound-Healing Assay

A human *ex vivo* wound-healing assay was performed to assess the healing promoting effects of the P_2_O_5_–CaO–MgO–Na_2_O PGF system (PGF-und) for a preliminary assessment. Human skin was obtained from patients undergoing reconstructive surgery at the Castle Hill Hospital, Cotthingham, United Kingdom. Skin was collected after the patients were fully informed, and a written patient consent was obtained following institutional guidelines and ethical approval (LREC: 17/SC/0220). *Ex vivo* wounding and whole mount staining were performed as described previously.^31^ Briefly, 2 mm partial thickness wounds were created in the center of 6 mm skin explants and cultured on a 0.45 μm nylon filter at the air-membrane interface. Growth media consisted of standard DMEM containing 10% FBS and 1% penicillin/streptomycin solution (Gibco). PGF-und was added in direct contact with the wound surface, and the wounds were cultured at 35 °C and 5% CO_2_ for 2 days before collecting in neutral buffered formalin. Wound explants were stained with antimouse keratin 14 antibody (clone: LL002; Abcam) to measure re-epithelialization and counterstained with 1 μg mL^–1^ 4′,6-diamidino-2-phenylindole (Thermo Fisher Scientific). Keratin 14 was detected using Alexa fluor 488-conjugated goat antimouse secondary antibody (Thermo Fisher Scientific). Wounds were imaged on a confocal laser scanning microscope (LSM 710, Carl Zeiss) using a 2.5X objective and 405 nm diode and 488 nm argon lasers. A one-way ANOVA with Tukey posthoc was performed on *ex vivo* data, with significance determined where *p < 0.05.

## 3 Results and Discussion

### 3.1 SEM Imaging and EDX Mapping

The composition of all PGFs was assessed via SEM equipped with an EDX detector. Their elemental composition expressed in terms of wt % of each element is reported in Table 1. Despite quantification of oxygen via EDX suffering from the fact that it is a light element, compositions in terms of oxides were calculated by considering the expected oxide stoichiometries as per ISO 22309:2011 guidance and previous works.^32^

**Table 1:** Composition of PGFs Expressed as wt % of Each Element, as Measured by EDX

sample name	P	Ca	Mg	Na	Fe	O
PGF-und	29.5 ± 0.2	7.6 ± 0.1	2.9 ± 0.1	7.6 ± 0.1		52.4 ± 0.3
PGF-Fe0.1	28.3 ± 0.2	8.2 ± 0.1	3.4 ± 0.1	5.9 ± 0.1	0.2 ± 0.1	54.0 ± 0.3
PGF-Fe0.5	29.6 ± 0.2	8.2 ± 0.1	3.3 ± 0.1	5.2 ± 0.1	0.7 ± 0.1	53.0 ± 0.3
PGF-Fe1	27.0 ± 0.2	7.3 ± 0.3	2.9 ± 0.1	4.7 ± 0.1	1.4 ± 0.2	56.7 ± 0.4
PGF-Fe2	25.2 ± 0.2	6.7 ± 0.1	3.1 ± 0.0	4.3 ± 0.0	2.4 ± 0.2	58.3 ± 0.3

**Figure 2:**
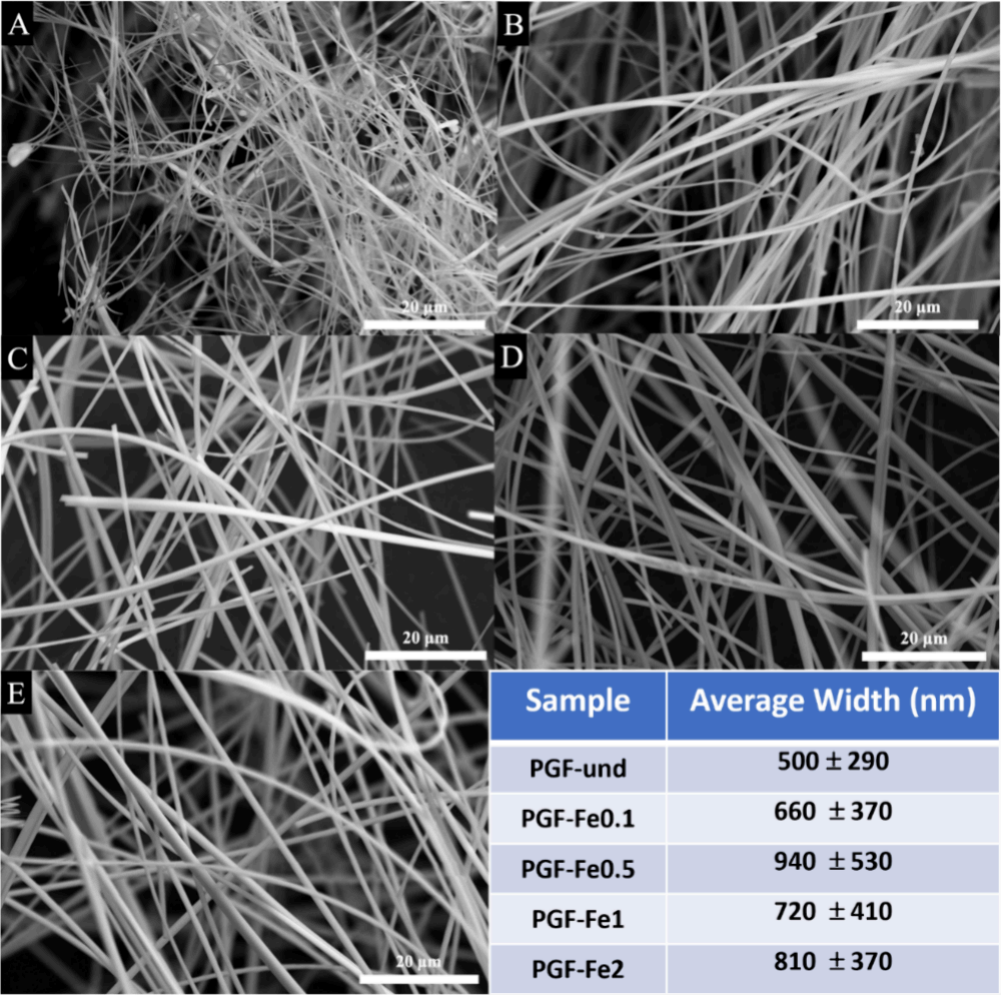
SEM images of (A) PGF-und, (B) PGF-Fe0.1, (C) PGF-Fe0.5, (D) PGF-Fe1, and (E) PGF-Fe2 and Table with average width ranges of PGF measured with MIPAR.

SEM images of all PGFs are shown in Figure 2. Bundles of PGFs can clearly be observed in all of the samples. An estimation of PGFs diameters was obtained from SEM images using the MIPAR image analysis software (Table in Figure 2).

**Figure 3:**
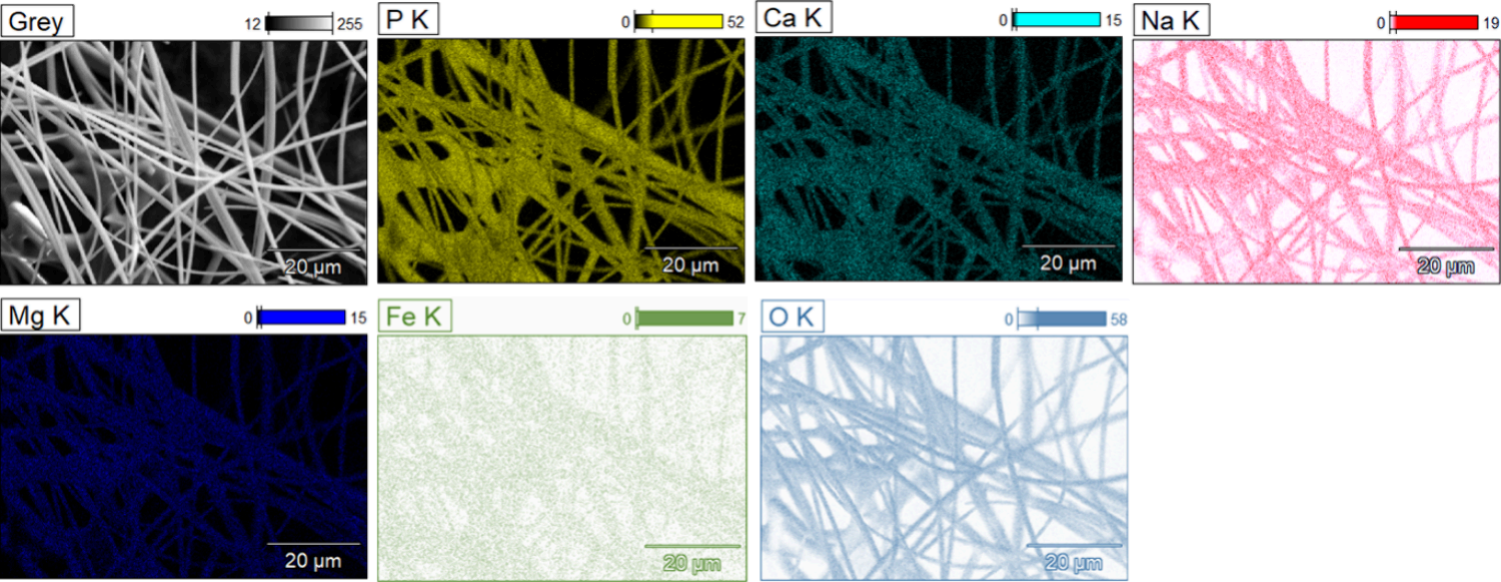
Representative SEM image of PGF-Fe2 (grey) and corresponding EDX mapping of all elements: P (yellow), Ca (cyan), Na (red), Mg (navy blue), Fe (green), and O (light blue).

The distribution of chemical species across the surface of all PGFs was investigated using EDX mapping. Results show that all elements are homogeneously distributed on the surface of PGFs, including iron. A representative EDX map of all elements for the sample PGF-Fe2 is shown in Figure 3.

### 3.2 XRD, FT-IR, and Raman Spectroscopy

**Figure 4:**
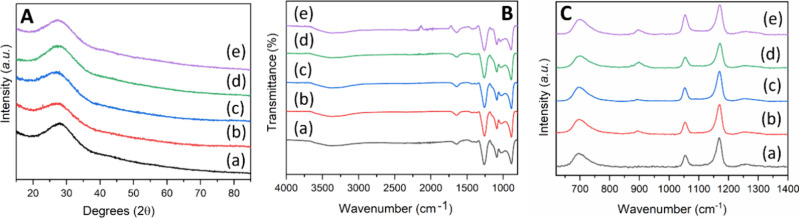
XRD patterns (A), FT-IR spectra (B), and Raman spectra (C) of (a) PGF-und, (b) PGF-Fe0.1, (c) PGF-Fe0.5, (d) PGF-Fe1, and (e) PGF-Fe2.

As shown in Figure 4A, XRD patterns of all PGFs do not present any Bragg peaks, indicating that they are all amorphous. The broad peak at at 2θ ∼ 28° is due to the amorphous phosphate network.

The structure of the amorphous phosphate network was investigated using FT-IR and Raman spectroscopies, as shown in Figure 4B and 4C, respectively. FT-IR and Raman bands were assigned according to previous studies on PGs.^15,33−35^ As the frequency of the vibrations of P–O bonds changes with the number of bridging oxygen atoms, the notation Q^*n*^ has been used, where *n* represents the number of bridging oxygens between phosphate units. Bands in both FT-IR and Raman spectra are quite broad, in agreement with the amorphous nature of PGFs. No significant differences are observed for both FT-IR and Raman spectra among all PGFs. The main FT-IR bands at ∼900 and ∼1250 cm^–1^ are due to Q^2^ bridging units, which confirm the presence of polyphosphate chains. Weaker bands are observed at approximately ∼1050 and ∼1150 cm^–1^, which correspond to the symmetric stretching (ν_s_) and asymmetric stretching (ν_as_) of the (PO_3_)^2–^ Q^1^ phosphate units, respectively. The band at ∼1050 cm^–1^ seems to be more pronounced for PGF-Fe2 samples, suggesting that Fe might disrupt the polyphosphate chains. The weak bands observed at ∼1625 and ∼3300 cm^–1^ are due to the H–O–H bending (δ) and the O–H stretching vibrations of residual water molecules, respectively.

In the Raman spectra, the broad band at ∼700 cm^–1^ can be ascribed to the symmetric stretching of in-chain P–O–P bridging oxygen groups ν_s_(P–O–P). The bands at ∼1150 cm^–1^ and the broader bands at ∼1250 cm^–1^ are due to symmetric (ν_s_) and asymmetric stretching (ν_as_) of the out-of-chain (O–P–O) units, respectively. The band at ∼1050 cm^–1^ is assigned to symmetric stretching (ν_s_) of the (PO_3_)^2–^_t_ species. The lower intensity band at ∼ 900 cm^–1^ could be ascribed to the asymmetric stretching ν_as_ of chain P–O–P groups, ν_as_(P–O–P). Interestingly, this band increases in intensity with iron content along with the band at ∼1050 cm^–1^. This observation suggests that the bands at 920 and 1050 cm^–1^ could have a contribution from the symmetric and asymmetric stretching vibrations of Fe–O–P bonds, respectively.^35^

### 3.3 Thermogravimetric Analysis

**Figure 5:**
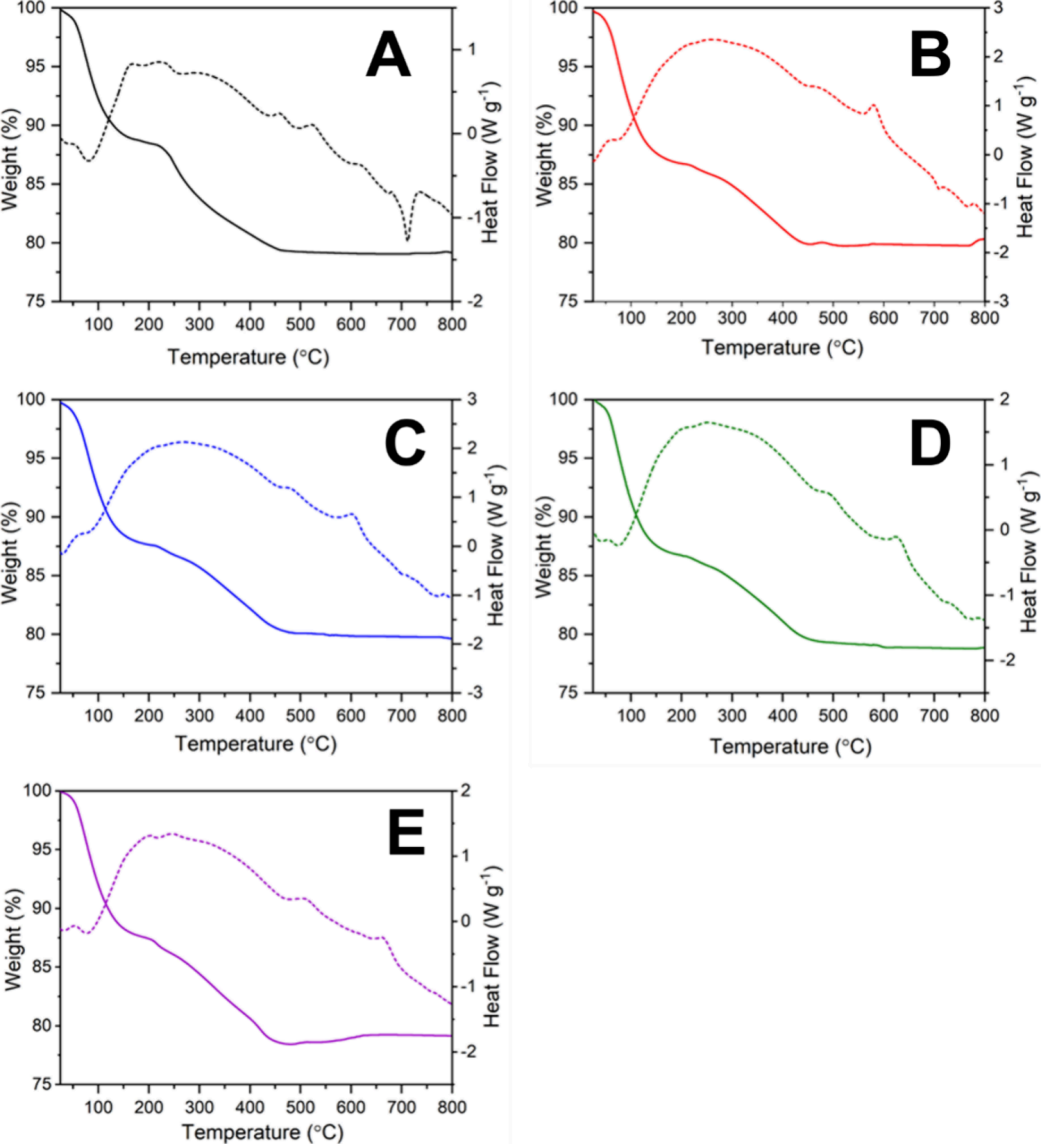
TGA (solid line) and DSC (dotted line) of (A) PGF-und, (B) PGF-Fe0.1, (C) PGF-Fe0.5, (D) PGF-Fe1, and (E) PGF-Fe2.

Weight loss and identification of thermal events upon heating were assessed on all PGFs using simultaneous DSC and TGA. TGA and DSC curves of all PGFs are shown in Figure 5, and the main weight losses and thermal events are reported in Table 2.

**Table 2:** Weight Loss % Obtained from TGA Plots; Crystallization Temperatures (*T*_c_) and Melting Temperatures (*T*_m_) Obtained from DSC Plots

	weight loss % (wt %)			thermal events		
sample	25–170 °C	170–450 °C	total	*T*_c_^1^ (°C)	*T*_c_^2^ (°C)	*T*_m_ (°C)
PGF-und	12.0	9.0	21.0	450	520	710
PGF-Fe0.1	13.0	7.5	20.5	480	580	709, 764
PGF-Fe0.5	12.5	7.5	20.0	490	600	704
PGF-Fe1	13.0	8.0	21.0	500	630	720, 770
PGF-Fe2	12.5	9.5	22.0	515	670	760

All PGFs show two distinct weight losses, the first from 25 to 170 °C (∼ 12–13%) and the second from 170 to 450 °C (∼ 7.5–9.5 wt %). The first weight loss is likely to be due to loosely bound water within the PGFs. This is in agreement with FT-IR analysis that shows some residual water in the PGF after ES. The second weight loss could be due to more tightly bound water and removal of nitrates.^32^

All samples show two exothermic peaks that could be ascribed to crystallization, the first in the range of 450–515 °C and the second in the range of 520–670 °C, with the crystallization temperature increasing with iron loading. This is agreement with the high thermal stability (low crystallization ability) of iron containing PG.^34,36^ PGF-und showed a sharp endothermic peak at ∼710 °C due to melting. Other PGFs show less pronounced *T*_m_ increase with iron loading.

### 3.4 *In Vitro* Dissolution Study

One of the main advantages of PGFs is their ability to dissolve in aqueous solutions, while releasing TMIs that can promote tissue regeneration and/or antibacterial activity.^37^

Wound healing occurs in different stages at different time scales: hemostasis (minutes-hours), inflammation (hours-days), proliferation (days-weeks), and remodelling (months). Each of the stages is affected differently by different ions *e.g.* Mg^2+^ and Ca^2+^ in the coagulation cascade (hemostasis).^22,38^

**Figure 6:**
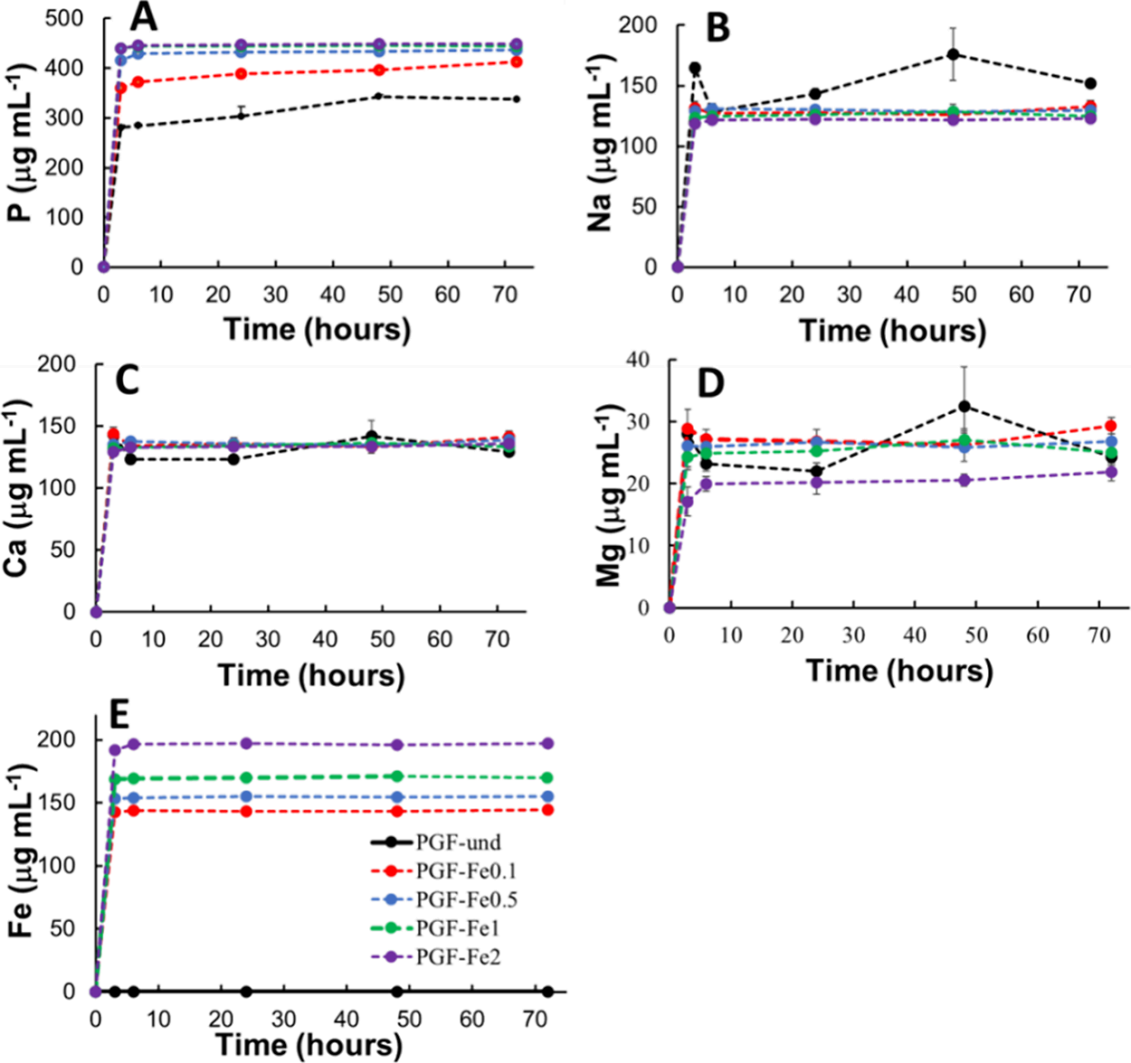
Release profiles of P (phosphate anions) (A), Na^+^ (B), Ca^2+^ (C), Mg^2+^ (D), and Fe^3+^ (E) after immersion in DI water up to 72 h from all PGFs. Error bars are ± SD (*n* = 3).

Given that the bioactivity of PGFs is strongly related to their release properties, an assessment of PGFs dissolution over time was performed using MP-AES for quantification of species released. The release profiles of P, Ca, Mg, Na, and Fe following immersion of the PGFs in DI water were measured at five different time points (3, 6, 24, 48, and 72 h) (Figure 6). It must be noted that MP-AES analyses provide information only on the total elemental concentrations but not about the species present in solution; however, for further discussion, it has been assumed that the free ionic species phosphate anions Ca^2+^, Na^+^, Mg^2+^, and Fe^3+^ were those released in DI water. As shown in Figure 6, all species show a high release in the first 3 h, followed by a much slower release. Details on the release after 24 h will be discussed in detail below. As expected, Fe^3+^ release clearly increases with its content in the PGF (∼143, 155, 170, and 197 μg mL^–1^ are released after 24 h by PGF-Fe0.1, PGF-Fe0.5, PGF-Fe1, and PGF-Fe2, respectively). Interestingly, a similar dependence is observed in the phosphate release (after 24 h, ∼303, 389, 432, and 447 μg mL^–1^ are released by PGF-und, PGF-Fe0.1, PGF-Fe0.5, and PGF-Fe1/PGF-Fe2, respectively), suggesting that the phosphate network is weakened by the addition of Fe^3+^, in agreement with FT-IR. A lower amount of Mg^2+^ seems to be released with increasing Fe^3+^ (∼20 μg mL^–1^ for PGF-Fe2 vs ∼27 μg mL^–1^ for PGF-und after 24 h), whereas the release of Ca^2+^ and Na^+^ are relatively independent of Fe^3+^ content. Ca^2+^ release is ∼123 μg mL^–1^ for PGF-und and ∼133 μg mL^–1^ for all PGF-Fe, whereas Na^+^ release is ∼143 μg mL^–1^ for PGF-und and ∼130–122 μg mL^–1^ for all PGF-Fe, the lowest value being released by PGF-2.

### 3.5 *In Vitro* Cytocompatibility and Antibacterial Activity

Biocompatibility of PGFs, was assessed by evaluating the viability of HaCaTs exposed to PGFs’ dissolution products obtained after a 24 h immersion time and incubated over a 24 h period.

**Figure 7:**
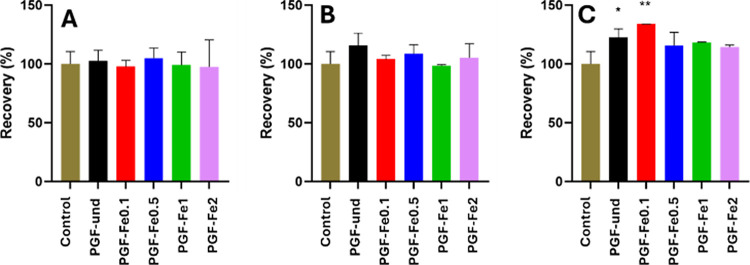
MTT assay analysis after 24 h incubation of HaCaTs exposed to 24 h dissolution products of PGFs: cell culture medium ratios of 1:100 (A), 3:100 (B), and 5:100 (C). Control = untreated cell medium. Error bars represent the standard deviation over triplicates (*p < 0.05; **p < 0.01; ***p < 0.001).

To investigate any dose-dependent effect of dissolution products on HaCaTs, three different ratios of the 24 h dissolution products to cell medium (DP-CM) were tested: 1:100, 3:100, and 5:100 respectively. Cell viability was quantitatively assessed using MTT analysis (Figure 7).

**Figure 8:**
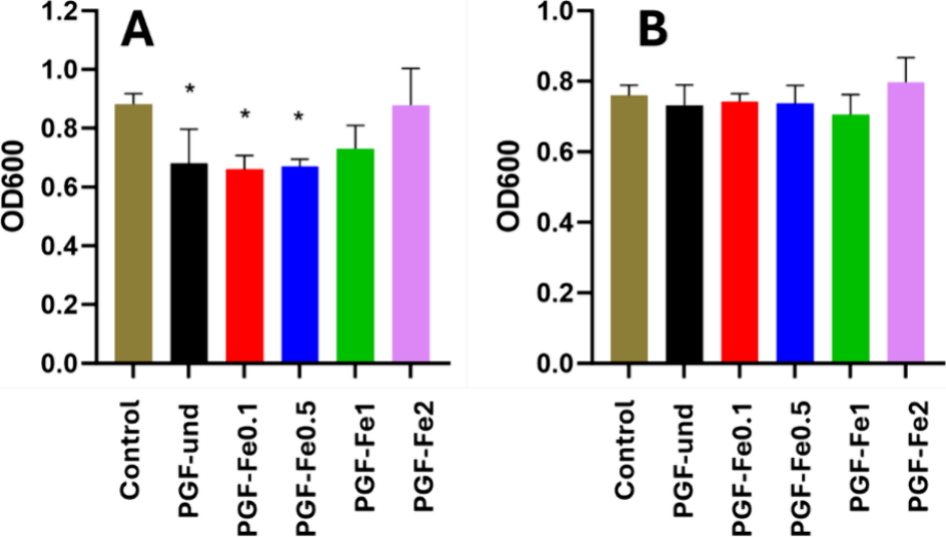
Antibacterial activity of PGFs against *E. coli* (A) and *S. aureus* (B) using a DP-CM ratio of 5:100. Error bars represent the standard deviation over triplicates (*p < 0.05; **p < 0.01; ***p < 0.001).

No cytotoxicity was observed on HaCaTs treated with PGFs regardless of the ratio of DP-CM or Fe loading. No statistically significant differences between the control and samples were observed for the DP-CM ratios of 1:100 and 3:100. However, the results of the MTT test performed using the DP-CM ratio 5:100 show some differences. In particular, sample PGF-Fe0.1 shows an increase in viability compared to the other samples which could be due to increased proliferation and/or metabolic activity. These results agree with previous studies on the effect of Mg and Fe ions on the proliferation of mammalian cells.^39^ As the DP-CM ratio of 5:100 gave the best MTT results, it was chosen as the optimum ratio to investigate the antibacterial activity of PGFs against *E. coli* and *S. aureus*, common bacterial strains found in wounds (Figure 8).

Three runs for each experiment were performed to validate the results. A statistically significant antibacterial effect can be observed on the cultures of **E. coli** treated with the dissolution products obtained after 24 h of immersion of PGF-und up to PGF-Fe0.5. The antibacterial effect of iron against **E. coli** has been previously reported.^40^ The antibacterial activity of PGF-und could be ascribed to a slight decrease in pH, as already observed in PGs prepared via MQ.^41^ As the antibacterial effect seems to decrease with dissolution products released from PGF-Fe1 and PGF-Fe2, a combined effect of pH and iron content could be responsible for the antibacterial activity. However, no antibacterial effect against *S. aureus* is observed. This could be due to the difference in the cell membrane structure of the two bacteria, which causes a different mechanism of action when positively charged metallic ions are used as antibacterial species. **E. coli** is a Gram-negative bacteria, whereas *S. aureus* is a Gram-positive bacteria. Previous antibacterial tests on coacervate PGFs containing copper ions showed higher resistance of *S. aureus* compared to **E. coli*.* This was ascribed to the fact that Gram-negative bacteria have a negatively charged coating on the membrane that makes them more sensitive to positively charged ions compared to Gram-positive bacteria.^42^

### 3.6 Human *Ex Vivo* Wound Model and Whole Mount Staining Data

**Figure 9:**
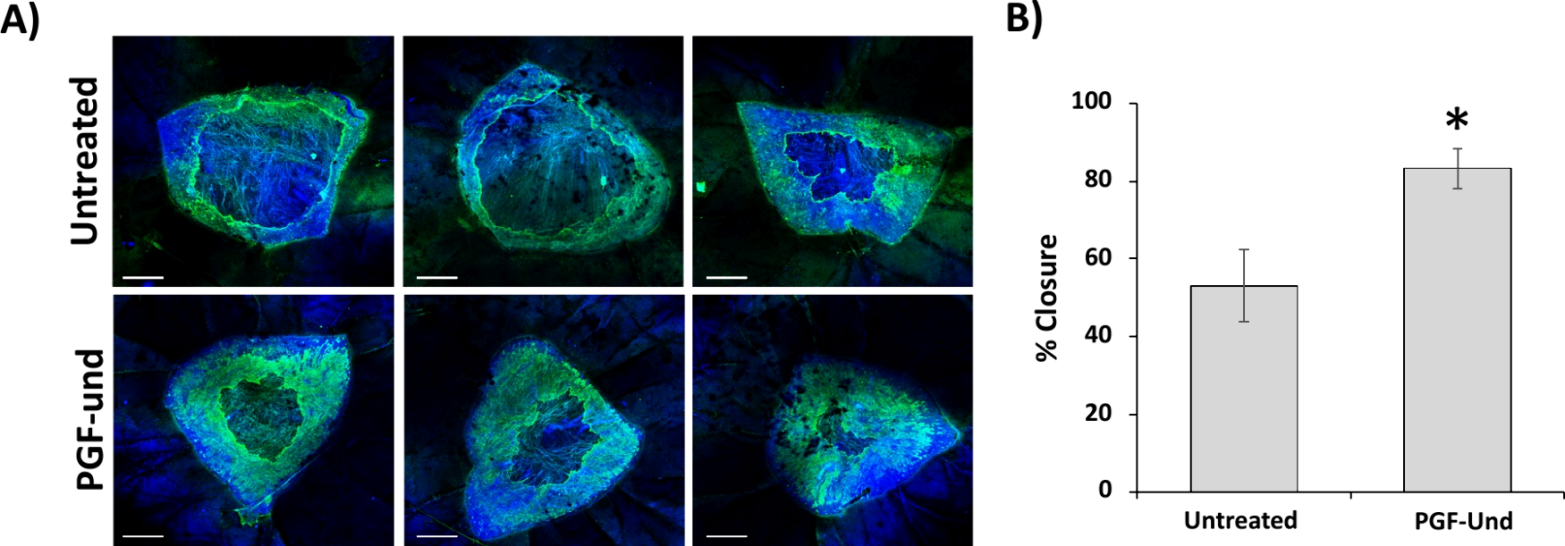
(A) Representative confocal images (A) and % closure (B) following 48 h of incubation in the human skin (untreated versus PGF-und). Scale bar = 500 μm. DAPI = nuclei. Alexa Fluor 488 = keratin 14 staining. Independent two-tailed *t* test where * = *p* < 0.05.

A preliminary test on the therapeutic wound-healing effects of the coacervate PGF-und were assessed by putting the sample directly in contact with human *ex vivo* skin wounds and assessing healing via whole mount staining (Figure 9). PGF-und were added directly to the growth media (6 mg/6 mL) and then placed on the biopsies (*n* = 3 per group). Growth media alone were used as the no-treatment control. The results show that PGF-und significantly accelerated wound closure (up to 84%) in this translationally relevant human skin model, therefore suggesting that PGF-und could show potential wound-healing efficacy in the clinic.

## 4 Conclusions

Phosphate-based coacervate gels in the system P_2_O_5_–CaO–MgO–Na_2_O–Fe_2_O_3_ (0, 0.1, 0.5, 1, and 2 wt % of Fe) were successfully electrospun for production of amorphous cotton-like bundles of fibers. EDX data showed a homogeneous distribution of all elements on the surface of all PGFs. Spectroscopic measurements showed that iron loading did not significantly affect the structure. However, FT-IR indicates a slight disruption of the polyphosphate chains with increasing Fe loading. Dissolution study in DI water showed that phosphate anion release decreases with increasing iron loading, meaning that the phosphate network is disrupted with this increase, which agrees with the FT-IR. Release of iron is dependent on its loading, as expected. *In vitro* cytocompatibility tested on HaCaTs in contact with 24 h dissolution products at three different ratios of DP-CM demonstrates lack of cytotoxicity at all ratios tested. In particular, using a DP-CM ratio of 5:100, PGF-und and PGF-Fe0.1 showed enhanced growth of HaCaTs and simultaneous inhibition of **E. coli** growth, making them very promising samples for wound healing and decreasing the risk of infection.

A preliminary test assessing the effects of PGF-und on human *ex vivo* wound repair demonstrated accelerated wound healing, with a significant increase in wound closure. Results show that PGFs prepared via the sustainable and facile route of coacervation have great potential as material for wound-healing applications, in particular for reducing the risk of **E. coli** bacterial infection.

## Supporting Information Available

Images of coacervate gel Coa-und, Coa-Fe1, and Coa-Fe2 (PDF)
