# Supplementary material for: Electrospun Polyphosphate Coacervate Glass Fibers in the System P2O5–CaO–MgO–Na2O–Fe2O3 for Wound Healing
Source: ACS Omega. 2025 Mar 17;10(11):10987–96. doi: 10.1021/acsomega.4c09366 (PMC11947787; doi:10.1021/acsomega.4c09366)
Supplement: Supplementary file 1 — ao4c09366_si_001.pdf [file ao4c09366_si_001.pdf]

SUPPORTING INFORMATION

Electrospun polyphosphate coacervate glass fibres in the system P_2O_5-CaO-MgO-Na_2O-Fe_2O_3 for wound healing

Jack Humphray, [a] Agron Hoxha, [a] Eveliny Tomás Nery, [a, b] Charlotte Berry, [a] Mónica Felipe-Sotelo, [a] Holly Wilkinson, [c,d] Matthew Hardman, [c,d] Jorge Gutiérrez-Merino [a, b] and Daniela Carta [a, *]

[a] *School of Chemistry and Chemical Engineering, University of Surrey, Guildford, GU2 7XH, United Kingdom.*

[b] *School of Biosciences and Medicine, University of Surrey, Guildford GU2 7XH, United Kingdom.*

[c] *Centre for Biomedicine, Hull York Medical School, University of Hull, Hull HU6 7RX, United Kingdom.*

[d] *Skin Research Centre, Hull York Medical School, University of York, York YO10 5DD, United Kingdom.*

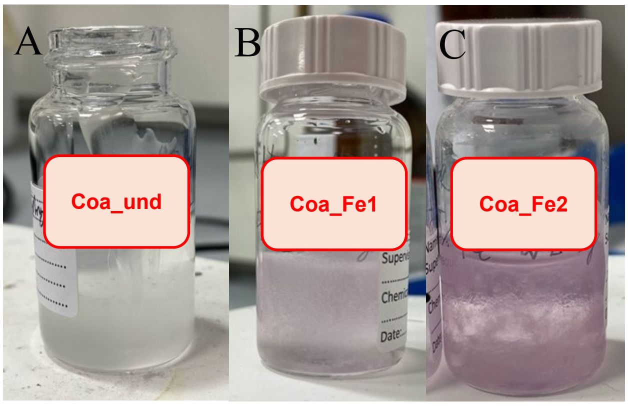

Figure SI-1. Images of coacervate gels: A) Coa_und; B) Coa_Fe1; C) Coa-Fe2
